# DNA methylation of the promoter region of *bnip3* and *bnip3l* genes induced by metabolic programming

**DOI:** 10.1186/s12864-018-5048-4

**Published:** 2018-09-17

**Authors:** Vincent Veron, Lucie Marandel, Jingwei Liu, Emilio J. Vélez, Olivier Lepais, Stéphane Panserat, Sandrine Skiba, Iban Seiliez

**Affiliations:** 1 INRA, Univ Pau & Pays de l’Adour, E2S UPPA, UMR1419 Nutrition Metabolism and Aquaculture, Aquapôle, F-64310 Saint-Pée-sur-Nivelle, France; 2 Department of Cell Biology, Physiology and Immunology, Faculty of Biology, University of Barcelona, Barcelona, Spain; 3 INRA, Univ Pau & Pays de l’Adour, UMR Ecobiop, Aquapôle, F-64310 Saint-Pée-sur-Nivelle, France

**Keywords:** Rainbow trout, Metabolic programming, bnip3, bnip3l, Hypoxia, Methionine, DNA methylation

## Abstract

**Background:**

Environmental changes of biotic or abiotic nature during critical periods of early development may exert a profound influence on physiological functions later in life. This process, named developmental programming can also be driven through parental nutrition. At molecular level, epigenetic modifications are the most likely candidate for persistent modulation of genes expression in later life.

**Results:**

In order to investigate epigenetic modifications induced by programming in rainbow trout, we focused on *bnip3* and *bnip3l* paralogous genes known to be sensitive to environmental changes but also regulated by epigenetic modifications. Two specific stimuli were used: (i) early acute hypoxia applied at embryo stage and (ii) broodstock and fry methionine deficient diet, considering methionine as one of the main methyl-group donor needed for DNA methylation. We observed a programming effect of hypoxia with an increase of *bnip3a* and the four paralogs of *bnip3l* expression level in fry. In addition, parental methionine nutrition was correlated to *bnip3a* and *bnip3lb1* expression showing evidence for early fry programming. We highlighted that both stimuli modified DNA methylation levels at some specific loci of *bnip3a* and *bnip3lb1*.

**Conclusion:**

Overall, these data demonstrate that methionine level and hypoxia stimulus can be of critical importance in metabolic programming. Both stimuli affected DNA methylation of specific loci, among them, an interesting CpG site have been identified, namely − 884 bp site of *bnip3a*, and may be positively related with mRNA levels.

**Electronic supplementary material:**

The online version of this article (10.1186/s12864-018-5048-4) contains supplementary material, which is available to authorized users.

## Background

Biotic and abiotic environmental changes encountered during critical early windows of developmental plasticity may exert a profound influence on physiological functions later in life [1]. This process named “developmental programming” has been proposed to prepare adult phenotypes to better cope with specific environment [2–4]. Over the past decades, developmental programming has been widely studied in mammals [5]. In contrast, the first studies on this topic in fish were initiated only recently and were mainly focused on nutritional and metabolic programming. In 2007, Vagner et al. showed higher and persistent delta 6 desaturase mRNA levels when European sea bass larvae were fed a low levels of n-3 HUFA diet [6]. In addition, an hyperglucidic diet applied at first feeding was found to lead to persistent changes in levels of transcripts related to glucose digestion and utilisation in zebrafish, gilthead seabream and rainbow trout juvenile [2, 3, 7, 8]. More recently long term effects of first feeding with plant-based diet have been tested successfully in trout, salmon and seabass, confirming the concept of nutritional programming in fish [9–12].

Most of these studies applied a nutritional stimulus at first exogenous feeding. However it is known that embryogenesis also represents a sensitive window of metabolic plasticity during which a stimulus may have a more efficient long-term effect [4, 13]. For instance, an acute hypoxic stimulus applied in rainbow trout at embryo stage modified mRNA levels of several glucose-metabolism related genes at first feeding [14]. Moreover, after the embryonic hypoxic stimulus, the glucose metabolism in liver and muscle of rainbow trout juvenile was impacted, confirming a long term programming effect of a very early stimulus [15]. Programming can also be driven through parental nutrition. For instance, feeding rainbow trout broodstock for 6 months with a methionine deficient diet affected the expression of genes involved into methionine metabolism in fry fed for 21 days [16]. Moreover, genes involved in gluconeogenesis and autophagy were also affected by parental methionine deficiency [17]. These data confirm that developmental programming with nutritional and non-nutritional stimuli applied directly at embryo stage or through broodstock nutrition can induce long-term metabolic programming in fish.

Different biological mechanisms have been shown to be involved in developmental programming. Clonal selection of adapted cells during differential proliferation of tissue cell type could explain physiological adaptations in later life [18]. At molecular level, epigenetic modifications are the likeliest candidates to consider in the context of nutrition and more particularly when working on programming. Indeed, these mechanisms are influenced by metabolic state as well as environmental changes [19] and can be maintained at long-term during cellular divisions at least through mitosis [20], even through meiosis. In mammals, many studies have focused on mechanisms involving epigenetic regulation of genes expression and their role in nutritional or metabolic programming [21–23]. By contrast, in fish, few data are available on epigenetic mechanisms which potentially underlie programming phenotypes and are mainly limited to modifications occurring at the whole epigenome level [15, 24, 25].

In order to deeper investigate epigenetic modifications induced by environmental programming and their involvement on resulting phenotypes in teleost at target gene level, it is essential to focus on a model gene known to be sensitive to environmental changes but also regulated by epigenetic modifications in a strong and repeatable way. In the present study, we focused on *bnip3* (bcl-2/E1B-19 K interacting protein 3), and *bnip3like* (also known as *nix*), two genes involved in mitochondrial mediated apoptosis and/or mitochondrial autophagy upon diverse cellular stress including hypoxia [26–28]. Changes in the expression of these two genes were previously demonstrated to be regulated by DNA methylation under hypoxic environment [29–32] but also to be associated to the activity of the one-carbon metabolism [33], the major metabolic supply route of methyl groups that are required for DNA and histone methylation.

Here, we aimed at studying epigenetic regulation of *bnip3* and *bnip3l* genes in rainbow trout subjected to two specific stimuli known to strongly affect these genes, hypoxia and methionine deprivation, in a context of metabolic programming. First, as *bnip3* and *bnip3l* genes can be induced by hypoxia [34–37], we studied the programming effect of an early acute hypoxic stimulus applied at embryo stage on the regulation of *bnip3* and *bnip3l* genes at fry stage. Secondly, regarding the role of methionine as methyl donor for epigenetic modifications [38, 39], we investigated the programming consequences on the regulation of *bnip3* and *bnip3l* genes on progeny of parents fed a methionine deficient diet during gametogenesis. This last step allowed investigating for the first time intergenerational programming at the epigenetic level in rainbow trout.

## Results

### Identification of *bnip3* and *bnip3l* genes in rainbow trout

Using the recent availability of the rainbow trout genome assembly [40], we identified two genes (Genoscope accession number: GSONMT00001151001 and GSONMT00082530001) sharing a high sequence homology (E-value >2e-09, Sigenae tblasn http://www.sigenae.org/) with the zebrafish *bnip3* available in Ensembl (ENST00000368636.8). Similarly, we found 4 genes (Genoscope accession number: GSONMT00078967001, GSONMT00064944001, GSONMT00079376001 and GSONMT00059781001) sharing a high sequence homology (E-value >2e-16) with the zebrafish *bnip3la* available in Ensembl (ENSDART00000035676.4).

**Fig. 1:**
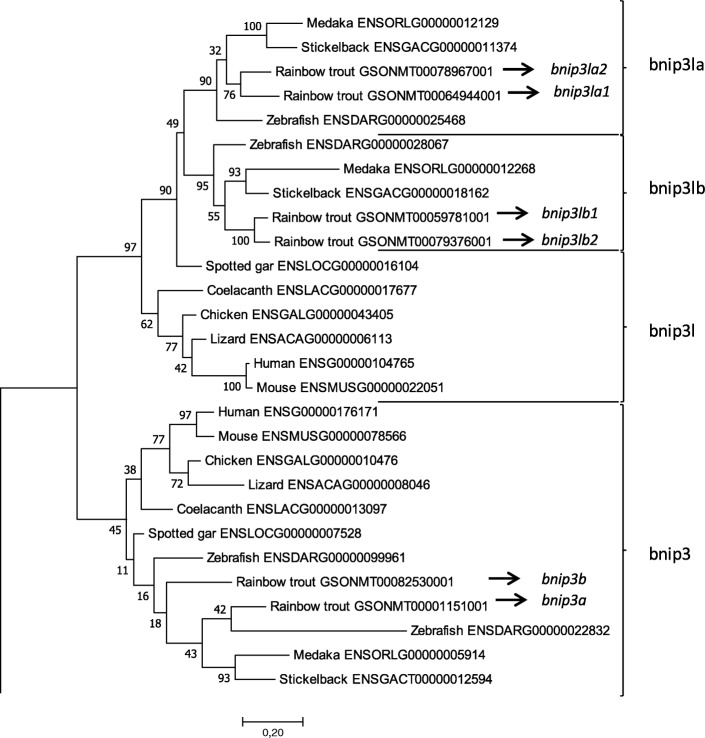
Phylogenetic tree, based on full length amino acid sequences, was built using the Maximum Likelihood Method (with Poisson model) using the Molecular Evolutionary Genetics Analysis (MEGA) software version 7.0 (Tamura 2013). The reliability of the interfered trees was estimated using bootstraps with 1000 replications. Accession numbers from Ensembl or Genoscope database are in brackets. Mammalian and teleost BNIP2 protein sequences were used to root the tree

In order to confirm the identity of the identified genes, a percentage identity matrix was established after alignment of the deduced amino acids (aa) sequences of these genes with those of *BNIP3* and *BNIP3L* from other vertebrate species including human, mouse, chicken, lizard, coelacanth spotted gar, zebrafish, medaka and stickleback (Additional file 1: Figure S1). The identity matrix showed that the deduced aa sequences of GSONMT00001151001 and GSONMT00082530001 shared a higher percent of homology with BNIP3 (mean of 57.6% of homology) than BNIP3L (mean of 49.1% of homology). Inversely, sequences GSONMT00078967001, GSONMT00064944001, GSONMT00079376001 and GSONMT00059781001 presented a higher homology with BNIP3L (mean of 59.8% of homology) than BNIP3 (mean of 50.6% of homology) when we compared trout sequences with other studied species. Accordingly, the phylogenetic analysis performed by the Maximum Likelyhood method (Poisson model, 1000 bootstraps) showed that the two trout sequences (GSONMT00001151001 and GSONMT00082530001), sharing the highest percent of homology with BNIP3, clustered with vertebrates BNIP3, while the four last sequences (GSONMT00078967001, GSONMT00064944001, GSONMT00079376001 and GSONMT00059781001) grouped together with vertebrates BNIP3L (Fig. 1). These results suggested that the two former trout genes (GSONMT00001151001 and GSONMT00082530001) are paralogous genes and co-orthologous to vertebrates *BNIP3*, while the four last genes (GSONMT00078967001, GSONMT00064944001, GSONMT00079376001 and GSONMT00059781001) are co-orthologous to vertebrates *BNIP3L*. Moreover, this phylogenetic tree also revealed that two trout *bnip3l* proteins (GSONMT00064944001 and GSONMT00078967001) rooted with teleosts *bnip3la* (defining them as *bnip3la1* and *bnip3la2*, respectively), while the two others (GSONMT00059781001 and GSONMT00079376001) were co-orthologous to *bnip3lb*, also identifying them as *bnip3lb1* and *bnip3lb2*, respectively.

**Fig. 2:**
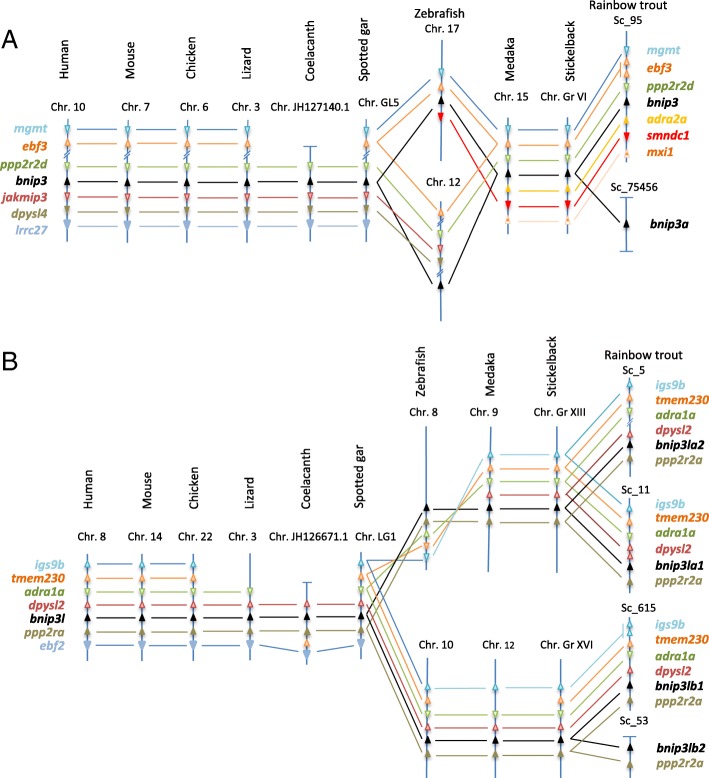
Synteny around *bnip3* (**a**) and *bnip3l* (**b**) *loci*. Syntenic analyses were conducted using Genomicus software (http://www.genomicus.biologie.ens.fr/genomicus-trout-01.01/)

We then performed a syntenic analysis to clarify the evolutionary history of *BNIP3* and *BNIP3L* in vertebrates. In all non-teleost species analysed here, *bnip3* was included in the *ppp2r2d-jakmip3-dpysl4-lrrc27* syntenic group highly conserved across species (Fig. 2a). Interestingly, a syntenic conservation of this region was found in two distinct chromosomes (17 and 12) of the zebrafish genome whereas only one syntenic region containing *bnip3* in medaka and stickleback was identified. Considering the newly sequenced rainbow trout genome, our syntenic analysis showed that GSONMT00001151001 (*bnip3a*) and GSONMT00082530001 (*bnip3b*) genes were localized on two distinct scaffolds (scaffold_75,456 and scaffold_95, respectively). The synteny around *bnip3b locus* was well conserved but the scaffold_75,456 bearing *bnip3a* was too short to provide relevant syntenic information. As regard *bnip3l*, our analysis showed a conservation of synteny in the vicinity of this *locus*. In all the studied vertebrates, *bnip3l* is included in *dpysl2-ppp2r2a* syntenic group (Fig. 2b). It should be noted that this conserved region bearing *bnip3l* is present in two distinct chromosomes in zebrafish, medaka and stickleback and four different scaffolds in rainbow trout.

### *bnip3* and *bnip3l* mRNA levels

**Fig. 3:**
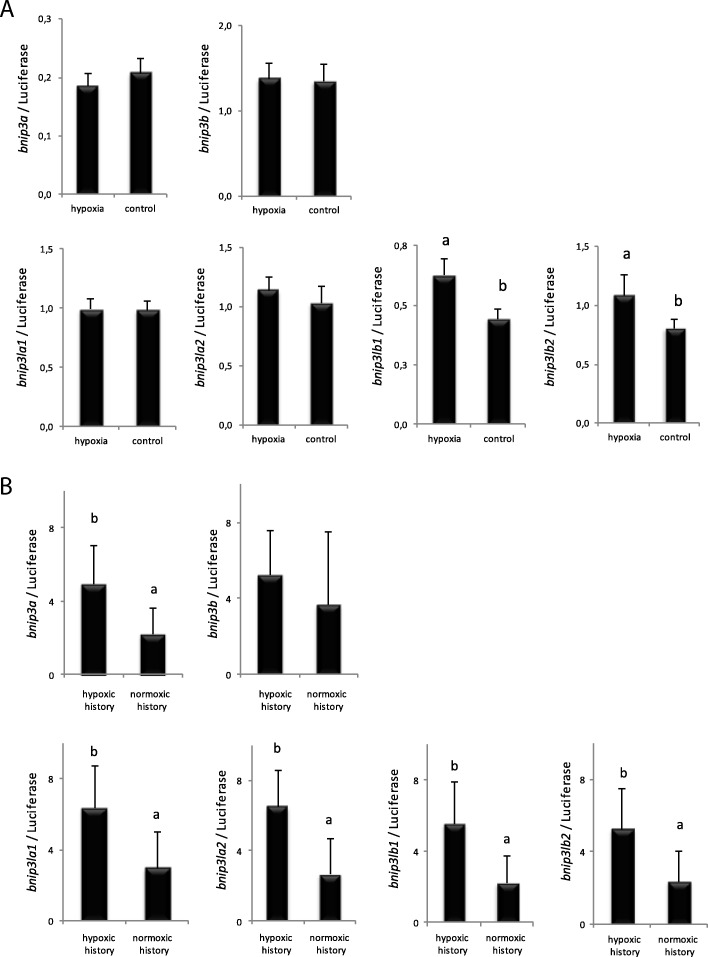
Effect of hypoxic stimulus at embryo stage on mRNA levels of *bnip3* and *bnip3l* genes in rainbow trout embryos (**a**) and fry (**b**). Data are expressed as mean ± SE (*n* = 6). Letters indicate significant differences between conditions (*p* < 0.05)

In mammals, hypoxia was shown to increase the expression of *bnip3* and *bnip3l* [27, 34–36, 41, 42]. Here, we aimed to determine whether the different *bnip3* and *bnip3l* paralogs identified in trout were affected by hypoxia as well as the possible existence of a “middle term” (until fry stage) programming effect of this stimulus on the expression of these genes. As shown in Fig. 3a, *bnip3lb1* and *bnip3lb2* were the only analysed genes exhibiting a significantly higher expression in embryos directly subjected to a 24 h hypoxic stress compared to control embryos kept at normoxic condition. However, surprisingly, at longer term (502 °D after hypoxia stimulus) fry from the hypoxic-embryos displayed significantly higher mRNA levels of not only *bnip3lb1* and *bnip3lb2* but also of *bnip3a*, *bnip3la1* and *bnip3la2* compared to the control group (Fig. 3b).

**Fig. 4:**
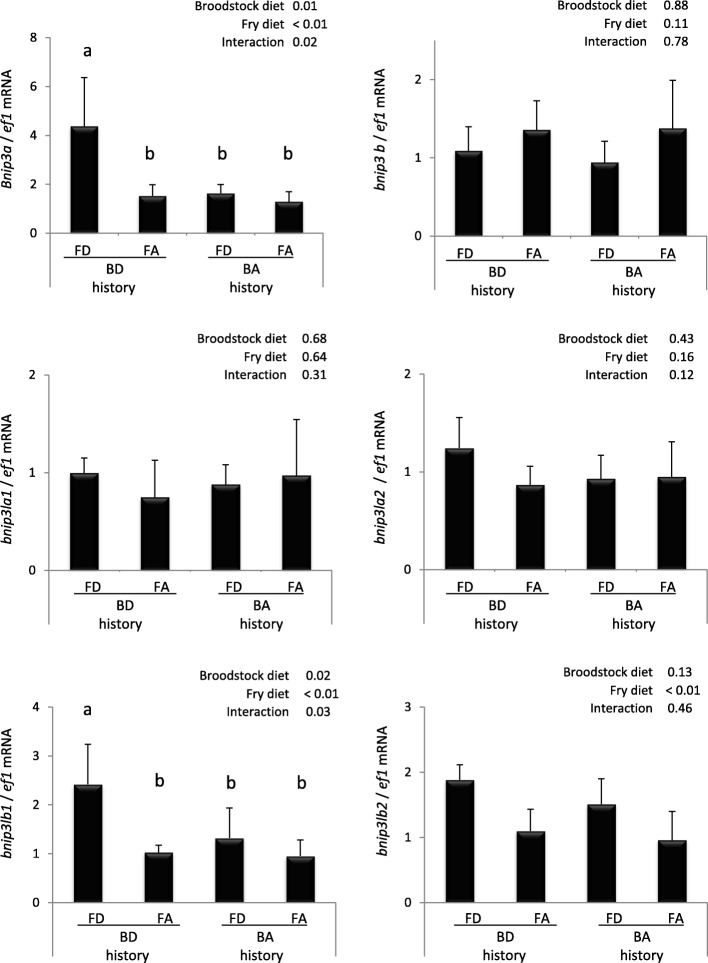
Effect of a 3 weeks Met deficiency in rainbow trout fry from BD and BA broodstock groups on mRNA levels of *bnip3* and *bnip3l* genes. BD and BA for Broodstock Deficiency and Adequate methionine diet, respectively. FD and FA for Fry Deficiency and Adequate methionine diet, respectively. Data are expressed as mean ± SE (*n* = 6). Letters indicate significant differences between conditions (*p* < 0.05)

Met emerged as a key factor in modulating the cellular availability of the main biological methyl donor S-adenosylmethionine (SAM) needed for all biological methylation reactions including DNA and histone methylation. As such, it represents a potential critical factor in nutritional programming. We therefore monitored the effect of feeding broodstock with a diet deficient in Met on the expression of identified *bnip3* and *bnip3l* genes in offspring subjected to different dietary Met levels. We found that *bnip3a* and *bnip3lb1* mRNA levels were induced by the Met deficiency in the diet only in offspring from Met deficient broodstock (BD-FD group significantly higher to the 3 other groups) (Fig. 4). We also found that Met deficiency applied in fry enhanced the expression of *bnip3lb2* whatever the broodstock diet. All the other studied genes did not display significant changes of their expression.

Together, these results support the possibility of programming the expression of some *bnip3* and *bnip3l* genes in rainbow trout by means of both early hypoxia stimulus and broodstock nutritional stimulus, thereby offering a good model to determine the underlying epigenetic mechanisms.

### *bnip3* and *bnip3l* promoters DNA methylation levels

We studied the DNA methylation of *bnip3* and *bnip3l* genes upstream region in fry of both experiments (hypoxia and Met deficiency). We focused on genes which expressions were affected by both the hypoxic stimulus and the Met deficient diet; namely *bnip3a* and *bnip3lb1*. Using target gene Next Generation Bisulfite Sequencing, we analysed DNA methylation at specific CpG sites along the 5′ upstream region of these two genes.

#### Effect of hypoxic history on DNA methylation level of bnip3a and bnip3lb1

**Fig. 5:**
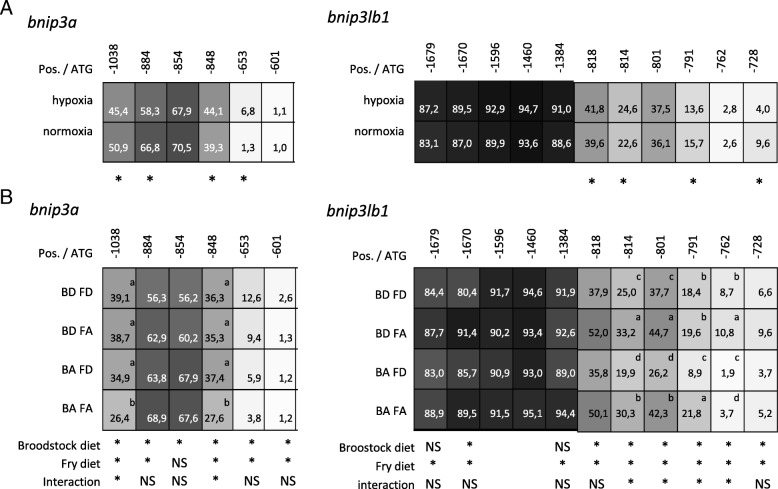
DNA methylation status of upstream region of *bnip3a* and *bnip3lb1* genes in fry with hypoxic history (**a**) and after different methionine levels in broodstock and fry diets (**b**). BD and BA for Broodstock Deficiency or Adequate methionine diet respectively. FD and FA for Fry Deficiency or Adequate methionine diet respectively. Data are expressed as percentage of methylation at each CpG site. Position of each CpG site is given related to ATG codon. Stars indicate significant differences between conditions (*p* < 0.01). NS, non-significant. Letters indicate significant differences between conditions (*p* < 0.01)

For both genes, we observed that the methylation level was low (from 0 to 3%) around the ATG predicted codon and until − 600 bp whatever the condition (data not shown). In the region upstream to − 600 bp, DNA methylation levels strongly increased to reach a maximum of 70.5% and 94.5% of methylated CpG for *bnip3* and *bnip3l,* respectively (Fig. 5a). Within this region, two CpG sites of *bnip3a* (at − 1038 and − 884 bp) and of *bnip3l* (at − 791 and − 728 bp) exhibited significantly lower methylation levels in fry with the hypoxic-history compared to their control counterparts. By contrast, the CpG sites at − 848 and − 653 bp of *bnip3a* and − 818 and − 814 bp of *bnip3lb1* presented higher methylation rates in fry with the hypoxia history.

#### Effect of broodstock and/or fry fed met deficient nutrition on the level of DNA methylation of bnip3a and bnip3lb1

As for hypoxic stimulation, we observed for both genes that the DNA methylation level was very low around the ATG codon (from 0 to 2.5%) and until − 600 bp (data not shown) whatever the condition. We also detected a strong increase of methylation levels in the regions upstream to − 600 bp. As regard *bnip3a*, we detected two trends. The first concerns the CpG sites at − 884 and − 854 bp, which showed an inhibitory effect of both the parental and fry methionine deficiency on DNA methylation levels (Fig. 5b). In contrast, the CpG sites at − 1038, − 848, − 653 and − 601 bp showed higher DNA methylation levels upon both parental and fry methionine deficiency. As regard *bnip3lb1*, the CpG sites downstream of − 1384 bp showed higher DNA methylation in fry from BD broodstock compared to those from BA broodstock (Fig. 5b). In contrast, the upstream CpG sites showed no effect of broodstock nutrition or even an opposite effect at − 1670 bp. In all cases (all analysed CpG sites), regardless of the broodstock diet, we observed a significantly lower DNA methylation levels in fry fed the methionine deficient diet.

## Discussion

In this work, we aimed at studying in rainbow trout epigenetic mechanisms underlying persistent changes of *bnip3* and *bnip3l* expression in a context of metabolic programming.

We first proceeded to the identification and characterization of the *bnip3* and *bnip3l* genes in trout. A previous phylogenetic analysis suggested that *bnip3* and *bnip3l* was restricted to metazoan lineage and both genes would have emerged from whole genome duplication (WGD) in the vertebrate ancestor [43]. The authors highlighted a low divergent selection pressure between both genes which was in accordance with their similar functions in apoptosis and mitophagy [26–28]. In the present study, we identified two *bnip3* paralogs in rainbow trout genome assembly [40]. *bnip3a* and *bnip3b*, clustered with *bnip3* teleost sequences on phylogenetic analysis. Moreover, we observed a high conserved synteny in the vicinity of *bnip3* genes between the studied species. This suggests that the two rainbow trout paralogs are co-orthologous to vertebrates *BNIP3* genes. It was not possible to study *bnip3a* synteny in rainbow trout due to a short scaffold length. However, both phylogenetic analysis and synteny suggest that the presence of two *bnip3* paralogs in rainbow trout genome may be due to the recent 4th salmonid specific WGD (named Ss4R) which occurred around 80–100 million years ago [40, 44, 45] suggesting that *bnip3a* and *bnip3b* were ohnologous genes.

We also identified four *bnip3l* paralogs in rainbow trout genome. Two sequences clustered with *bnip3la* and the remaining two with *bnip3lb* teleost sequences on the established phylogenetic tree. Furthermore, in all studied vertebrates we observed conservation of genes synteny in the vicinity of *bnip3l locus*. This suggests that rainbow trout *bnip3l* paralogs are co-orthologous to vertebrates *bnip3l* genes. This syntenic group is present in two distinct chromosomes in teleosts (zebrafish, medaka and stickleback) and in a unique copy in holostei (represented here by the spotted gar). Phylogenetic and syntenic data supported that the teleost-specific WGD (Ts3R) gave rise to *bnip3la* and *bnip3lb* paralogs [46–50]. Our results also showed that *bnip3la1* and *bnip3la2* identified in rainbow trout are co-orthologs to zebrafish, medaka and stickelback *bnip3la*. Similarly, *bnip3lb1* and *bnip3lb2* are co-orthologous with zebrafish, medaka and stickleback *bnip3lb*. Regarding the phylogenic and syntenic analysis, the duplication of rainbow trout *bnip3la* and *bnip3lb* genes into *bnip3la1* and *bnip3la2*, and *bnip3lb1* and *bnip3lb2*, respectively, arose at the Ss4R. Therefore, existence of four *bnip3l* paralogs in rainbow trout could be explained by two rounds of WGD, Ts3R and Ss4R. Overall, these gene duplication events of *bnip3l* and *bnip3* offered an interesting model to study potential divergences in both the function and the expression of the related paralogs.

Numerous studies in mammals reported that early developmental stages can be critical windows of metabolic plasticity [1]. In this regard, early life events such as nutritional or environmental changes can affect growth, health and metabolic status later in life [3, 4]. Here, we show in rainbow trout that hypoxia (applied to embryo for 24 h) can induce, at least until fry stage, the expression of *bnip3a* and the four *bnip3l* paralogs. Previously, genes involved in gluconeogenesis pathway and glucose transport were reported to be affected at fry stage in same experimental conditions [15]. Therefore we confirm that hypoxia act as a developmental programming stimulus.

Our results also showed that hypoxia lead to significant changes in DNA methylation levels of upstream regions of both *bnip3a* and *bnip3lb1*. DNA methylation is often associated with gene silencing [51, 52]. It has been observed in mammalian cancer cells, especially in hypoxic microenvironment, that *BNIP3* expression is repressed by promoter aberrant hypermethylation [29, 31, 53–56]. This specific epigenetic alteration allows cancer cells to escape to BNIP3 and BNIP3L proapoptotic activity. Silencing of *BNIP3* expression was associated with methylation of the hypoxia-responsive element (HRE) site that in turn inhibited the binding of hypoxia-inducible factor 1 (HIF1α) to the BNIP3 promoter [56]. In our study, hypoxia induced lower methylation level at CpG sites − 1038 and − 884 of *bnip3a* and − 791 and − 728 of *bnip3lb1.* Interestingly, *bnip3a* displays two possible HRE at sites − 1038 and − 884 (data not shown), fitting consensus sequence of HRE1 and HRE2 (R-CGTC and R-CGTG, respectively), described previously [34, 57–59], and making possible a direct link between the hypomethylation at these specific *loci* and the upregulation of *bnip3a* expression observed in fry from the hypoxic-embryos. However, the higher methylation levels observed in neighbouring CpG sites (− 848 and − 653 for *bnip3a* and − 818 and − 814 for *bnip3lb1*) highlights the complexity of epigenetic mechanisms at play in the control of gene expression [60].

Similarly to early developmental events, parental nutrition can have an impact on growth potential, heath and metabolism of the offspring [1, 61]. Here, we observed higher expression of both *bnip3a* and *bnip3lb1* genes in the BD-FD group compared to other fry, supporting the existence of an early fry programming by the mean of parental nutrition. Previously, in the same experimental conditions, Seiliez et al. also reported that it was possible to drive nutritional programming in fish through parental nutrition. Met deficiency in broodstock diet affected gene expression of fatty acid, cholesterol synthesis and autophagy in fry [17]. Overall, our results confirmed that Met level can be of critical importance in metabolic programming and prompted us to investigate epigenetic mechanisms at play in the effect observed.

In mammals, early Met nutrition was shown to affect DNA methylation later on life by controlling the one-carbon metabolism [62, 63]. More recently, in zebrafish, parental deficiency of one-carbon metabolism-related metabolites was observed to affect DNA methylation levels at some specific genes *loci* of the offspring [64]. In the present study, we clearly demonstrated that both broodstock and early fry Met nutrition affected the methylation of several CpG sites of *bnip3a* and *bnip3lb1* genes. However, as for hypoxia, the effect of Met deficiency (applied to both broodstock and fry) differs between the different CpG, making difficult to depict a real picture of mechanism at play in this event. It should be noted, however, that the methylation levels at the CpG site − 884 of *bnip3a* (which exhibit an HRE-like sequence) may be positively related with the mRNA levels of *bnip3a*.

In the future, additional investigations by ChIP and promoter fusion analysis approaches should be done to better understand function of key CpG identified here. Gene regulation is complex and most of the time results from the combination of different mechanisms. For instance, histone modifications have a major impact on expression regulation by opening or condensing chromatin which enable or not transcription factor binding. Another interesting subject will be to determine whether the effects observed at fry stage persist until adult stage for both stimuli applied.

## Conclusion

Collectively, we highlighted in this study modifications of DNA methylation levels of *bnip3a* and *bnip3lb1* genes in a context of metabolic programming. An interesting zone has been identified, namely − 884 bp site of *bnip3a*, which would deserve additional functional analyses in the future.

## Methods

### Ethical issues and approval

The INRA facilities are authorized for animal experimentation under French regulations (B 29–277-02). The experiments were carried out in accordance with the Guidelines of the National Legislation on Animal Care of the French Ministry of Research (Decree N_2001–464, May 29, 2001). The project was approved by the French National Consultative Ethics Committee (reference number 2015112018112159 and 201511201756973).

### In silico analysis

The genoscope database (http://www.genoscope.cns.fr/trout) was used to identify *bnip3* and *bnip3l* related genes in the rainbow trout genome using BLAST analysis. Sequences are available under the accession numbers GSONMT00001151001 and GSONMT00082530001 for *bnip3* and GSONMT00064944001, GSONMT00078967001, GSONMT00059781001 and GSONMT00079376001 for *bnip3l*, respectively.

Ensembl database (http://www.ensembl.org/index.html) was also used to collect amino acids deduced sequences of *bnip3* and *bnip3l* for human, mouse, chicken, lizard, spotted gar, medaka, stickleback, zebrafish and coelacanth.

Protein alignment and the percentage Identity Matrix established with amino acids deduced sequences were performed using MUSCLE software (http://www.ebi.ac.uk/Tools/msa/muscle/).

Phylogenetic tree, based on full length amino acid sequences, was built using the Maximum Likelihood method (with Poisson model) and confirmed by Neighbor-Joining method (data not shown) using the Molecular Evolutionary Genetics Analysis (MEGA) software version 7.0 [65]. The reliability of the interfered trees was estimated using the bootstrap method with 1000 replications. Mammalian and teleost bnip2 protein sequences were used to root the tree.

Syntenic analyses were conducted using Genomicus software (http://www.genomicus.biologie.ens.fr/genomicus-trout-01.01/) to confirm the identity of rainbow trout genes.

### Experimental designs

All fish used in this study were acquired from our INRA experimental fish farm facilities (Lées-Athas, France).

#### Hypoxia stimulus

**Fig. 6:**
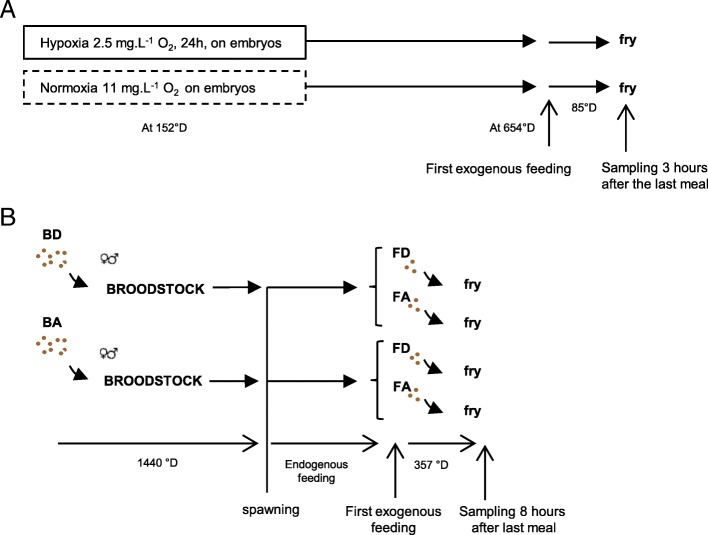
Experimental design. **a** 24 h hypoxia stimulus (2.5 mg.mL^− 1^ O_2_) was applied to embryos (152 °D). Fish were fed their first meal at 654 °D. After 85 °D of feeding, fry were sampled 3 h after last meal. A normoxia group was used as control. **b** Broodstock fish were fed for 1440 °D with either a methionine deficient diet (BD) or a control (Adequate, BA) diet. Males and females of each feeding group were then crossed and the obtained fry were fed with either the FD (Deficient) or the FA (Adequate) diet for 357 °D from the first exogenous feeding

The experimental design was previously described by Liu and collaborators [14] where the efficiency of the hypoxic stimulus was validated. Rainbow trout oocytes were fertilised and reared at 8 °C in 12 separate tanks in the INRA facilities (Lées-Athas, France). A 24 h hypoxia stimulus (2.5 mg.l^− 1^ dissolved oxygen, ~ 22%) was applied in 6 tanks to embryos at 152 degree days (°D) (Fig. 6a). The remaining 6 tanks of embryos were kept under normoxic conditions (11 mg.l^− 1^ dissolved oxygen) as a control group. After the 24 h hypoxic stimulation, embryos were kept under normoxic conditions. After hatching, alevins were reared with natural spring water in tanks at 18 °C under natural photoperiod. At 654 °D fry fed their first meal (Additional file 2: Table S1). After 85 °D (5 days) of feeding, fry were sampled 3 h after last meal by terminal anesthetization by bathing in benzocaine (30 mg/l then a bath at 60 mg/l) and were then frozen in liquid nitrogen and stored at − 80 °C until analysis. Fry which received the hypoxia at embryonic stage will be called later “fry with hypoxic-history”.

#### Methionine deficiency stimulus

The experimental design was detailed in Fontagné-Dicharry et al. [16] and summarized in Fig. 6b. Briefly, female and male rainbow trout broodstock were reared in our experimental fish farm facilities (INRA, Lées-Athas, France), in a flow-through rearing system supplied with natural spring water (8 °C) under natural photoperiod (April to October). Fish were randomly divided into two dietary groups (35 fish/group) that were fed for 1440 °D (6 months) before spawning one of the two plant-based diet providing two dietary methionine (Met) levels, set at 0.5 or 1% of the diet in the deficient (BD) or adequate (BA) diets, respectively (Additional file 2: Tables S2 and S3). Broodstock growth, relative fecundity, egg size and egg amino acid composition were analyzed and reported in Fontagné-Dicharry et al. [16]. Approximately 3000–5000 eggs collected for each female of each nutritional group were fertilized with a pool of sperm (collected from 5 to 8 males fed the corresponding diet). Embryos were reared at INRA experimental facilities at Lées-Athas in 8 °C stream water until the swim-up fry stage at the age of 528 °D; hatching occurred at the age of 352 °D. Following this, the swim-up fry were transferred to the experimental facilities at INRA Donzacq (France) and randomly distributed into 12 circular tanks (50 l; 400 fish/tank) supplied with natural spring water (17 °C) under natural photoperiod. The first-feeding fry were fed during 357 °D (3 weeks) a fry diet containing Met in adequate (FA) or deficient (FD) amounts (*n* = 3 tank/diet/broodstock group) (Additional file 2: Tables S2 and S3). After 3 weeks, *n* = 9 fish/tank were killed 8 h after the last meal by terminal anesthetization by bathing in benzocaine (30 mg/l then a bath at 60 mg/l) and were then frozen in liquid nitrogen and stored at − 80 °C before further mRNA and DNA analyses.

### RNA extraction and rtPCR

Total RNA was extracted from the whole body of fry (six fry were extracted per condition) using the TRIzol reagent method (Invitrogen, Carlsbad, CA) with Precellys 24 (Bertin technologies, Montigny le Bretonneux, France) following Trizol manufacturer’s instructions. 1 μg of total RNA was reverse transcribed in duplicate with SuperScript III RNAse H Reverse Transcriptase kit (Invitrogen) and random primer (Promega) according to the manufacturer’s instructions. mRNA levels were assayed using LightCycler 480 SYBR Green I Master (Roche Diagnostics, Neuilly sur Seine, France), 0.4 mM final of each primer, 2 μl of cDNA in a total volume of 6 μl. A LightCycler 480 II thermocycler (Roche) was used. The qPCR protocol was initiated by an initial denaturation step at 95 °C for 10 min followed by 45 cycles of a two steps amplification programme (15 s at 95 °C and 10 s at 60 °C). For each sample RT and qPCR was run in duplicate, negative controls for RT and qPCR were included.

For hypoxia stimulus, luciferase control RNA (Promega) was used, 10 pg per 1.9 mg of fry added to each sample to allow data normalisation as previously described [66]. For Met deficient diet stimulus, transcripts were normalized using Elongation Factor 1α (*ef1α*) as reference gene transcript following the Pfaffl method [67]. Primer sequences used to amplify *bnip3* and *bnip3l* paralogs are presented in Table 1.

**Table 1:** Primers used for mRNA levels measurement

Gene	Primer (5′ to 3′)	Tm in °C
*bnip3a*	F: CCTGTGACAGTCCTCCGAGA	60
	R: CCACTTCACGTCTCCGTTCT	
*bnip3b*	F: GAGAACAACCCACCAAAGGA	60
	R: GTATATCCCCAGGCCAACTG	
*bnip3la1*	F: CAAACTCCACCACACCCTCT	60
	R: CTGATCTGGACTGGGAGGTC	
*bnip3la2*	F: GGAGAGTCAGGCCCCTCAG	61
	R: TCCTGATCTGGACTGGAAGG	
*bnip3lb1*	F: GAACAACGGAGACGCTGGA	61
	R: GGTGGAGGTAGACTGGGACA	
*bnip3lb2*	F: GCTGTATCAGAGAACAACGGACTA	60
	R: CATGCTGAGCGTCCAGTAGA	

### Target gene DNA methylation study

#### DNA extraction

Whole body fry were digested in 5 ml of ice cold extraction buffer (125 mM NaCl, 10 mM EDTA, 0.5% SDS, 4 M urea, 10 mM tris-HCl, pH = 8) with 80 μg.ml^− 1^ final of proteinase K (P6556, Sigma-Aldrich) overnight at 37 °C under agitation (250 rpm). Six replicates per conditions were performed.

After overnight digestion, 5 ml of phenol chloroform isoamyl alcohol (25:24:1) was added to each sample. After mixing by inverting tubes, samples were centrifuged 15 min at 10000 g at room temperature. Aqueous phases were kept and 675 μl 5 M NaCl and 5 ml 100% ice-cold ethanol were added. After a 15 min centrifugation at 10000 g and 4 °C, pellets were washed with 1 mL 75% ice-cold ethanol and then centrifuge again 15 min, 10,000 g at 4 °C. Pellets were dried and re-suspend in H_2_O DNase free and treated containing 2 μg of RNase (R4642, Sigma) for 1 h at 37 °C. Quality of DNA was checked on 1% agarose gel and quantification was made using Nanodrop (Thermofisher, USA).

#### Analysis of target gene methylation by targeted next-generation bisulfite sequencing

The upstream sequence of *bnip3* located on the scaffold 75,456 (GSONMT00001151001) and *bnip3l* located on the scaffold 615 (GSONMT00059781001) were assessed.

The sequence GSONMT00001151001 of *bnip3* started 513 base pairs before the ATG codon so we sequenced the 5’UTR until − 1148 bp from ATG using the Universal GenomeWalker 2.0 (Clontech Laboratories, USA) and following manufacturer’s instructions. Sanger sequencing was done by Eurofins Genomics (Paris) to identify 5’UTR region. Then, MethPrimer software (http://www.urogene.org/cgi-bin/methprimer/methprimer.cgi) [68] was used to design primers (Table 2) targeting *bnip3* and *bnip3l* upstream regions. Each extracted DNA was bisulfite converted using EZ DNA Methylation-Gold Kit (D5005, Zymo Research, USA) following manufacturer’s instructions. In order to prevent any PCR artefact, three PCR replicates were run for each bisulfite converted DNA. Advantage 2 polymerase Mix (639,206, Clontech laboratories, USA) was used for amplification. PCR conditions were 94 °C for 2 min, and 40 cycles at 94 °C for 25 s, melting temperature (T_m_ mentioned for each primers set in Table 2) for 1 min and 72 °C for 2 min followed by a final step of 7 min at 72 °C. For each condition, all 10 amplicons (5 for each studied gene) run in triplicates were pooled. Libraries were generated using KAPA library preparation Kit (KAPA Biosystems, USA) at EpigenDx (Hopkington, USA). Sequencing was performed at EpingeDx on Ion Torrent PGM using 314 Chip kit v2. The NGS QC Toolkit v2.3.3 [69] was used to trim data removing part of the sequences with a quality score lower than 18 followed by a removal of reads smaller than 35 nucleotides using Bowtie 2 [70] using gene sequences in silico bisulfite converted as a reference. Alignment BAM files were then sorted by target and condition using BAM tools [71] split function. Sorted reads were analyzed in BiQ Analyzer HT [72] setting parameters at 100% of the read length, and bisulfite conversion efficiency ≥98% and lower cutoff at 20 reads per CpG site analyzed. The methylation level of each sampled cytosine was estimated as the number of reads reporting a C, divided by the total number of reads reporting a C or T. Data are expressed as percentage of methylation at each CpG site. Positions of CpG sites were determined from ATG site.

**Table 2:** Primers used for target gene DNA methylation study

Gene	Location from ATG	Primers (5′ to 3′)	Tm in °C
*bnip3a*	− 1085 / -762	F: TGATGGAATATTTAGTTTTTAGTAGGATAA	57
		R: TCCAAACCATCCAAAACTATTTAA	
	− 717 / -498	F: TTTTATGGATGGAGGAAATATTTGT	57
		R: TAAACAACTCTCTAAACTATTAAC	
	−513 / -283	F: TAGAGAGTTGTTTTATATAGGAAAA	57
		R: ATCACTCACTAATATATTCATTAATC	
	− 383 / -120	F: TTTGAATTTGTTTAATAGAAATTTT	54
		R: ATATTATTCTAATACCTCTAAATTA	
	−202 / + 43	F: TGTTTTGGTAGTTTAGTGTT	58
		R: CCTGCAAATTTTCCT	
*bnip3lb1*	− 1735 / -1358	F: AAAGAGATAGATATTTTGAGATTTGTTATA	57
		R: TAATAAATAAATTCCACTTCACTCC	
	− 1435 / -1038	F: TGAAGAATTGTTATGAAAGAGGTAATGT	57
		R: TCTCCAAAACCTATATTTACCATAAAC	
	− 867 / -590	F: GGGTATTTTAAAATTTTATTAATTTTTTATT	57
		R: ACACTTATTTAACAATTTAACACTTATTTA	
	− 594 / -222	F: GTGTGAGGTGAATTTAAGTTGT	59
		R: AATAATCCAATTCTTTAATAACAAAAACA	
	−252 / + 26	F: TGTTTTTGTTATTAAAGAATTGGATTATTT	58
		R: TCAACTACAACAACAACTTCAAAC	

### Statistical analysis

Statistical analysis of mRNA measure. For hypoxia stimulus, normality of distributions was assessed using the Shapiro-Wilk test and data were then analysed by Krustal-Wallis non-parametric test followed by Tuckey’s test as *post hoc* analysis. For Met deficient diet stimulus, effects of broodstock diet, effects of fry diet and interaction of both factors were tested by two-way ANOVA. *post hoc* tests were performed using Tukey’s multiple mean comparisons.

Statistical analysis of gene target methylation measure. For hypoxia stimulus, data were analyzed by a binomial generalized linear model followed by a Tukey test as a *post-hoc* analysis in order to test statistical significance of difference of DNA methylation observed between conditions at each CpG. For Met deficient diet stimulus, effects of broodstock diet, effects of fry diet and interaction of both factors were analysed by a binomial generalized linear model followed by Chi-square test. Tukey test as a post-hoc analysis were run to test difference between each conditions.

All statistical calculations were made using R software (v3.1.0)/R Commander Package [73].

## Additional files

Additional file 1: **Figure S1.** Protein alignment and the percentage Identity Matrix established with amino acids deduced sequences were performed using MUSCLE software (http://www.ebi.ac.uk/Tools/msa/muscle/). RT for rainbow trout. In brackets is given gene identity, 3 for BNIP3 and 3 l for BNIP3L. (PDF 167 kb)

Additional file 2: **Table S1.** Formulation and proximate composition of diet used in hypoxia stimulus [14]. **Table S2.** Formulation and proximate composition of diet used in methionine deficiency stimulus [16]. **Table S3.** Analyzed amino acid composition of the diets as g/100 g dry feed used in methionine deficiency stimulus [16]. (DOCX 24 kb)

## Abbreviations

BNIP3: bcl-2/E1B-19 K interacting protein3
Chip: Chromatin ImmunoPrecipitation
HUFA: Highly unsaturated fatty acids
Met: methionine
