# Supplementary material for: DNA methylation of the promoter region of bnip3 and bnip3l genes induced by metabolic programming
Source: BMC Genomics. 2018 Sep 17;19:677. doi: 10.1186/s12864-018-5048-4 (PMC6142374; doi:10.1186/s12864-018-5048-4)
Supplement: Supplementary file 1 — Figure S1. Protein alignment and the percentage Identity Matrix established with amino acids deduced sequences were performed using MUSCLE software (http://www.ebi.ac.uk/Tools/msa/muscle/). RT for rainbow trout. In brackets is given gene identity, 3 for BNIP3 and 3 l for BNIP3L. (PDF 167 kb) [file 12864_2018_5048_MOESM1_ESM.pdf]

Suppl. Fig. 1

	Coelacanth (3L)	Human (3L)	Mouse (3L)	Chicken (3L)	Lizard (3L)	Medaka (3La)	RT : GSONMT00078967001 (3La2)	RT : GSONMT00064944001 (3La1)	Stickelback (3Lb)	Zebrafish (3Lb)	RT : GSONMT00059781001 (3Lb1)	RT : GSONMT00079376001 (3Lb2)	Medaka (3La)	Stickelback (3La)	Spotted gar (3L)	Zebrafish (3La)	Zebrafish (4)	RT : GSONMT00001151001 (3a)	Chicken (3)	Lizard (3)	Human (3)	Mouse (3)	Zebrafish (3)	Spotted gar (3)	Coelacanth (3)	RT : GSONMT00082530001 (3b)	Medaka (3)	Stickelback 3
Coelacanth (3L)	100.00	71.08	69.61	75.12	74.15	51.56	64.47	64.57	65.48	66.32	65.64	64.95	67.51	69.04	72.59	66.50	34.88	50.90	57.36	53.85	59.59	59.02	53.97	59.69	57.44	51.11	48.17	49.19
Human (3L)	71.08	100.00	97.25	86.85	85.65	48.72	61.81	56.25	63.37	64.77	64.62	62.89	63.45	63.64	70.71	65.15	42.01	52.73	61.14	54.29	55.67	59.78	55.91	61.70	58.85	55.37	50.00	48.35
Mouse (3L)	69.61	97.25	100.00	85.45	85.58	49.23	61.31	57.03	62.87	64.25	64.62	62.89	62.94	63.13	69.70	64.65	42.01	52.73	61.66	54.29	56.16	59.78	55.38	61.70	59.38	55.37	50.50	47.80
Chicken (3L)	75.12	86.85	85.45	100.00	93.46	48.97	64.14	59.84	62.19	66.15	62.89	63.73	67.35	66.50	72.59	65.48	38.46	53.66	60.31	55.00	56.50	59.44	56.45	61.17	59.90	54.80	51.52	50.55
Lizard (3L)	74.15	85.65	85.58	93.46	100.00	48.97	62.63	57.48	63.00	65.10	63.40	62.69	66.33	65.99	72.08	65.48	38.46	53.66	61.34	53.57	53.57	60.00	55.38	61.17	59.90	54.24	51.76	50.00
Medaka (3La)	51.56	48.72	49.23	48.97	48.97	100.00	53.99	60.54	68.22	62.44	67.46	67.79	50.93	51.16	60.78	54.42	28.57	34.16	41.80	36.84	40.54	43.35	37.36	40.22	40.96	38.15	35.14	36.52
RT : GSONMT00078967001 (3La2)	64.47	61.81	61.31	64.14	62.63	53.99	100.00	79.63	66.20	66.51	67.57	65.75	70.31	70.13	76.56	73.33	32.76	45.24	52.04	50.00	49.74	53.33	49.21	51.31	49.74	50.00	45.55	46.49
RT : GSONMT00064944001 (3La1)	64.57	56.25	57.03	59.84	57.48	60.54	79.63	100.00	57.82	60.56	61.69	60.93	70.81	69.94	75.89	75.16	35.78	38.05	46.03	40.00	47.93	48.65	42.15	44.26	46.83	44.64	36.07	38.46
Stickelback (3Lb)	65.48	63.37	62.87	62.19	63.00	68.22	66.20	57.82	100.00	77.62	80.56	79.53	62.84	62.10	75.48	64.38	34.10	48.19	56.70	56.52	54.69	60.11	53.48	56.08	53.89	51.12	43.75	47.54
Zebrafish (3Lb)	66.32	64.77	64.25	66.15	65.10	62.44	66.51	60.56	77.62	100.00	79.15	79.33	66.98	68.08	78.89	69.95	36.26	46.91	56.84	58.21	57.92	60.34	53.01	55.14	53.97	49.15	44.09	45.05
RT : GSONMT00059781001 (3Lb1)	65.64	64.62	64.62	62.89	63.40	67.46	67.57	61.69	80.56	79.15	100.00	91.07	64.60	64.76	75.36	66.82	34.30	49.09	55.96	56.20	55.91	58.19	53.23	56.38	55.21	51.98	44.62	46.70
RT : GSONMT00079376001 (3Lb2)	64.95	62.89	62.89	63.73	62.69	67.79	65.75	60.93	79.53	79.33	91.07	100.00	65.18	64.29	75.73	66.36	34.50	49.39	56.77	56.83	56.22	58.52	54.59	56.68	55.50	50.57	45.95	47.51
Medaka (3La)	67.51	63.45	62.94	67.35	66.33	50.93	70.31	70.81	62.84	66.98	64.60	65.18	100.00	82.55	74.18	70.31	32.76	47.62	54.59	50.71	51.85	53.89	51.32	52.88	52.82	52.22	45.50	45.95
Stickelback (3La)	69.04	63.64	63.13	66.50	65.99	51.16	70.13	69.94	62.10	68.08	64.76	64.29	82.55	100.00	77.57	74.03	34.48	48.81	55.10	52.86	52.11	54.44	51.85	53.40	53.85	52.78	46.84	47.57
Spotted gar (3L)	72.59	70.71	69.70	72.59	72.08	60.78	76.56	75.89	75.48	78.89	75.36	75.73	74.18	77.57	100.00	78.97	36.99	52.35	58.59	57.75	56.77	59.34	56.02	58.03	56.35	52.20	48.44	49.20
Zebrafish (3La)	66.50	65.15	64.65	65.48	65.48	54.42	73.33	75.16	64.38	69.95	66.82	66.36	70.31	74.03	78.97	100.00	36.21	45.24	53.06	52.86	52.63	53.89	50.79	52.88	52.31	49.44	44.74	45.41
Zebrafish (4)	34.88	42.01	42.01	38.46	38.46	28.57	32.76	35.78	34.10	36.26	34.30	34.50	32.76	34.48	36.99	36.21	100.00	41.72	45.71	40.77	47.13	48.21	44.00	47.70	45.20	50.89	44.12	43.20
RT : GSONMT00001151001 (3a)	50.90	52.73	52.73	53.66	53.66	34.16	45.24	38.05	48.19	46.91	49.09	49.39	47.62	48.81	52.35	45.24	41.72	100.00	67.44	64.34	65.87	66.05	72.35	69.82	69.19	63.35	65.66	65.85
Chicken (3)	57.36	61.14	61.66	60.31	61.34	41.80	52.04	46.03	56.70	56.84	55.96	56.77	54.59	55.10	58.59	53.06	45.71	67.44	100.00	78.38	82.72	85.03	70.62	79.08	77.00	63.24	61.26	61.05
Lizard (3)	53.85	54.29	54.29	55.00	53.57	36.84	50.00	40.00	56.52	58.21	56.20	56.83	50.71	52.86	57.75	52.86	40.77	64.34	78.38	100.00	73.79	72.22	66.43	68.06	69.86	57.66	59.85	58.39
Human (3)	59.59	55.67	56.16	56.50	53.57	40.54	49.74	47.93	54.69	57.92	55.91	56.22	51.85	52.11	56.77	52.63	47.13	65.87	82.72	73.79	100.00	91.98	70.00	80.95	81.35	61.54	58.21	62.64
Mouse (3)	59.02	59.78	59.78	59.44	60.00	43.35	53.33	48.65	60.11	60.34	58.19	58.52	53.89	54.44	59.34	53.89	48.21	66.05	85.03	72.22	91.98	100.00	70.11	82.51	80.21	61.93	62.15	64.77
Zebrafish (3)	53.97	55.91	55.38	56.45	55.38	37.36	49.21	42.15	53.48	53.01	53.23	54.59	51.32	51.85	56.02	50.79	44.00	72.35	70.62	66.43	70.00	70.11	100.00	78.87	71.94	67.20	62.43	62.77
Spotted gar (3)	59.69	61.70	61.70	61.17	61.17	40.22	51.31	44.26	56.08	55.14	56.38	56.68	52.88	53.40	58.03	52.88	47.70	69.82	79.08	68.06	80.95	82.51	78.87	100.00	85.35	67.93	64.74	67.72
Coelacanth (3)	57.44	58.85	59.38	59.90	59.90	40.96	49.74	46.83	53.89	53.97	55.21	55.50	52.82	53.85	56.35	52.31	45.20	69.19	77.00	69.86	81.35	80.21	71.94	85.35	100.00	65.59	64.58	65.45
RT : GSONMT00082530001 (3b)	51.11	55.37	55.37	54.80	54.24	38.15	50.00	44.64	51.12	49.15	51.98	50.57	52.22	52.78	52.20	49.44	50.89	63.35	63.24	57.66	61.54	61.93	67.20	67.93	65.59	100.00	66.31	67.74
Medaka (3)	48.17	50.00	50.50	51.52	51.76	35.14	45.55	36.07	43.75	44.09	44.62	45.95	45.50	46.84	48.44	44.74	44.12	65.66	61.26	59.85	58.21	62.15	62.43	64.74	64.58	66.31	100.00	76.56
Stickelback (3)	49.19	48.35	47.80	50.55	50.00	36.52	46.49	38.46	47.54	45.05	46.70	47.51	45.95	47.57	49.20	45.41	43.20	65.85	61.05	58.39	62.64	64.77	62.77	67.72	65.45	67.74	76.56	100.00
