# Supplementary material for: DNA methylation of the promoter region of bnip3 and bnip3l genes induced by metabolic programming
Source: BMC Genomics. 2018 Sep 17;19:677. doi: 10.1186/s12864-018-5048-4 (PMC6142374; doi:10.1186/s12864-018-5048-4)
Supplement: Supplementary file 2 — Table S1. Formulation and proximate composition of diet used in hypoxia stimulus [14]. Table S2. Formulation and proximate composition of diet used in methionine deficiency stimulus [16]. Table S3. Analyzed amino acid composition of the diets as g/100 g dry feed used in methionine deficiency stimulus [16]. (DOCX 24 kb) [file 12864_2018_5048_MOESM2_ESM.docx]

| Ingredient (g/100g diet) |  |
| --- | --- |
| Fish meal^1^ | 91.2 |
| Fish oil^2^ | 4.8 |
| Starch^3^ | 0.0 |
| Vitamin mix^5^ | 1.0 |
| Mineral mix^6^ | 1.0 |
| Alginate^7^ | 2.0 |
| *Analytical composition* |  |
| Dry matter (DM,% diet) | 93.4 |
| Crude protein (% DM) | 63.0 |
| Crude lipids (% DM) | 16.1 |
| Gross energy (kJ g^-1^ DM) | 22.4 |
| Ash (% DM) | 15.6 |
| Carbohydrates (% DM) | 2.4 |

**Table S1**. **Formulation and proximate composition of diet used in hypoxia stimulus (Liu et al. 2017).**

^1^ Sopropeche, Boulogne-sur-Mer, France; ^2^ Sopropeche, Boulogne-sur-Mer,France; ^3^ gelatinized corn starch (Roquette, Lestrem, France); ^5^ supplying (kg^−1^ diet): 60 IU DL-α-tocopherol acetate, 5 mg sodium menadione bisulphate, 15,000 IU retinyl acetate, 3000 IU DL-cholecalciferol, 15 mg thiamine, 30 mg riboflavin, 15 mg pyridoxine, 0.05 mg vitamin B_12_, 175 mgnicotinic acid, 500 mgfolic acid, 1000 mginositol, 2.5 mg biotin, 50 mg calcium panthothenate and 2000 mg choline chloride; ^6^ supplying (kg^−1^diet): 2.15 g calcium carbonate (40% Ca), 1.24 g magnesium oxide (60% Mg), 0.2 g ferric citrate, 0.4 mgpotassium iodide (75% I), 0.4 g zinc sulphate (36% Zn), 0.3 g copper sulphate (25% Cu), 0.3 g manganese sulphate (33% Mn), 5 g dibasic calcium phosphate (20% Ca, 18% P), 2 mg cobalt sulphate, 3 mg sodium selenite (30% Se), 0.9 g potassium chloride and 0.4 g sodium chloride; and ^7^ Louis François, Marne-la-Vallée, France.

| Ingredient (g/100g diet) | BD | BA | FD | FA |
| --- | --- | --- | --- | --- |
|  |  |  |  |  |
| Fish soluble protein concentrate ^a^ | 4.0 | 4.0 | 5.0 | 5.0 |
| Fish oil ^a^ | 13.6 | 13.6 | 7.0 | 7.0 |
| Soybean protein concentrate ^a^ | 27.0 | 27.0 | 24.0 | 24.0 |
| Faba bean protein concentrate ^b^ | 19.0 | 19.0 | 27.5 | 27.5 |
| White lupin meal ^c^ | 11.0 | 11.0 | 18.0 | 18.0 |
| Dehulled pea meal ^b^ | 6.0 | 6.0 | 7.0 | 7.0 |
| Wheat gluten ^d^ | 4.0 | 4.0 | - | - |
| Whole wheat ^e^ | 8.0 | 8.0 | - | - |
| Soybean lecithin ^f^ | - | - | 3.0 | 3.0 |
| Carophyll® pink ^g^ | 0.03 | 0.03 | - | - |
| CaHPO4·2H2O ^e^ | 3.55 | 3.55 | 2.7 | 2.7 |
| Vitamin premix ^h^ | 1.0 | 1.0 | 2.0 | 2.0 |
| Mineral premix ^i^ | 1.0 | 1.0 | 2.0 | 2.0 |
| L-lysine ^j^ | 0.32 | 0.32 | 0.3 | 0.3 |
| L-glutamic acid ^k^ | 1.5 | 1.0 | 1.5 | 1.0 |
| DL-methionine ^l^ | 0.0 | 0.5 | 0.0 | 0.5 |
|  |  |  |  |  |
| *Analytical composition* |  |  |  |  |
|  |  |  |  |  |
| Dry matter (DM, %) | 95.6 | 95.9 | 95.9 | 95.8 |
| Crude protein (% DM) | 43.9 | 43.8 | 48.2 | 48.2 |
| Total lipid (% DM) | 16.7 | 16.0 | 14.7 | 13.4 |
| Gross energy (kJ/g DM) | 22.4 | 22.2 | 23.0 | 23.0 |
| Ash (% DM) | 7.9 | 8.0 | 8.0 | 8.1 |
|  |  |  |  |  |

**Table S2. Formulation and proximate composition of diet used in methionine deficiency stimulus (Fontagné-Dicharry et al. 2017).**

^a^ CPSP Special G, crude fish oil and Estrilvo from Sopropêche (Wimille, France).

^b^ Fabaqua 55 and Primatex from Sotexpro (Berméricourt, France).

^c^ Farilup 500 from Terrena (Martigné-Ferchaud, France).

^d^ Roquette (Lestrem, France).

^e^ Sud-Ouest Aliment (Haut-Mauco, France)

^f^ Louis François (Croissy-Beaubourg, France).

^g^ DSM (Basel, Switzerland), contained 8% astaxanthine.

^h^ Vitamin premix (IU or g/kg premix): retinyl acetate, 500,000 IU; cholecalciferol, 250,000 IU; DL-α-tocopheryl acetate, 5,000 IU; sodium menadione bisulfate, 1 g; thiamin-HCl, 0.1 g; riboflavin, 0.4 g; niacin, 1 g; D-calcium pantothenate, 2 g; pyridoxine-HCl, 0.3 g; D-biotin, 20 mg; folic acid, 0.1 g; cyanocobalamin, 1 mg; L-ascorbyl-2-polyphosphate, 5 g; *myo*-inositol, 30 g; choline, 100 g. All ingredients were diluted with α-cellulose.

^i^ Mineral mixture (g/kg premix): CaHPO4·2H2O, 500; CaCO3, 215; Mg(OH)2, 124; KCl, 90; NaCl, 40; FeSO4 · 7H2O, 20; ZnSO4 · 7H2O, 4; MnSO4·H2O, 3; CuSO4·5H2O, 3; NaF, 10; KI, 0.04; Na2SeO3, 0.03; CoCl2 · 6H2O, 0.02. All ingredients were diluted with α-cellulose.

^j^ Ajinomoto-Eurolysine (Paris, France).

^k^ Acros (Geel, Belgium).

^l^ Evonik (Essen, Germany).

BD for broodstock deficient methionine diet, BA for broodstock adequate, FD for fry deficient, FA for fry adequate.

| Diet | BD | BA | FD | FA |
| --- | --- | --- | --- | --- |
|  |  |  |  |  |
| *Essential amino acids ^a^* |  |  |  |  |
|  |  |  |  |  |
| Arginine | 3.14 | 3.23 | 4.66 | 4.84 |
| Histidine | 0.98 | 1.00 | 1.15 | 1.18 |
| Isoleucine | 1.66 | 1.67 | 2.22 | 2.22 |
| Leucine | 2.97 | 3.04 | 3.59 | 3.58 |
| Lysine | 2.53 | 2.60 | 2.78 | 2.69 |
| Methionine | 0.51 | 1.04 | 0.57 | 1.18 |
| Phenylalanine | 1.88 | 1.95 | 2.36 | 2.46 |
| Threonine | 1.54 | 1.58 | 1.67 | 1.71 |
| Valine | 1.77 | 1.77 | 2.44 | 2.45 |
|  |  |  |  |  |
| *Non-essential amino acids* |  |  |  |  |
|  |  |  |  |  |
| Alanine | 1.67 | 1.73 | 1.71 | 1.67 |
| Aspartic acid + Asparagine | 4.02 | 4.12 | 3.85 | 3.69 |
| Cysteine | 0.60 | 0.59 | 0.55 | 0.47 |
| Glutamic acid + Glutamine | 9.09 | 8.86 | 7.39 | 6.77 |
| Glycine | 1.76 | 1.81 | 2.15 | 2.18 |
| Proline | 2.14 | 2.21 | 2.00 | 2.01 |
| Serine | 2.07 | 2.15 | 2.34 | 2.32 |
| Tyrosine ^b^ | - | - | 1.93 | 1.94 |
|  |  |  |  |  |

**Table S3. Analyzed amino acid composition of the diets as g/100 g dry feed used in methionine deficiency stimulus (Fontagné-Dicharry et al. 2017).**

^a^ Tryptophan was not analyzed.

^b^ Tyrosine was not determined in broodstock diets.

BD for broodstock deficient methionine diet, BA for broodstock adequate, FD for fry deficient, FA for fry adequate.
